# Automatic Annotation of Subsea Pipelines Using Deep Learning

**DOI:** 10.3390/s20030674

**Published:** 2020-01-26

**Authors:** Anastasios Stamoulakatos, Javier Cardona, Chris McCaig, David Murray, Hein Filius, Robert Atkinson, Xavier Bellekens, Craig Michie, Ivan Andonovic, Pavlos Lazaridis, Andrew Hamilton, Md Moinul Hossain, Gaetano Di Caterina, Christos Tachtatzis

**Affiliations:** 1 Department of Electronic and Electrical Engineering, University of Strathclyde, Glasgow G11XW, UK; j.cardona-amengual@strath.ac.uk (J.C.); c.mccaig@strath.ac.uk (C.M.); robert.atkinson@strath.ac.uk (R.A.); xavier.bellekens@strath.ac.uk (X.B.); c.michie@strath.ac.uk (C.M.); i.andonovic@strath.ac.uk (I.A.); andrew.w.hamilton@strath.ac.uk (A.H.); gaetano.di-caterina@strath.ac.uk (G.D.C.); christos.tachtatzis@strath.ac.uk (C.T.); 2 N-Sea, RS 4301 Zierikzee, The Netherlands; d.murray@n-sea.com (D.M.); h.filius@n-sea.com (H.F.); 3 Department of Engineering and Technology School of Computing and Engineering, Huddersfield HD1 3DH, UK; P.Lazaridis@hud.ac.uk; 4 School of Engineering and Digital Arts, University of Kent, Canterbury, Kent CT2 7NT, UK; M.Hossain@kent.ac.uk

**Keywords:** visual inspection, sub-sea pipeline survey, multi-label image classification, deep learning, transfer learning

## Abstract

Regulatory requirements for sub-sea oil and gas operators mandates the frequent inspection of pipeline assets to ensure that their degradation and damage are maintained at acceptable levels. The inspection process is usually sub-contracted to surveyors who utilize sub-sea Remotely Operated Vehicles (ROVs), launched from a surface vessel and piloted over the pipeline. ROVs capture data from various sensors/instruments which are subsequently reviewed and interpreted by human operators, creating a log of event annotations; a slow, labor-intensive and costly process. The paper presents an automatic image annotation framework that identifies/classifies key events of interest in the video footage viz. exposure, burial, field joints, anodes, and free spans. The reported methodology utilizes transfer learning with a Deep Convolutional Neural Network (ResNet-50), fine-tuned on real-life, representative data from challenging sub-sea environments with low lighting conditions, sand agitation, sea-life and vegetation. The network outputs are configured to perform multi-label image classifications for critical events. The annotation performance varies between 95.1% and 99.7% in terms of accuracy and 90.4% and 99.4% in terms of F1-Score depending on event type. The performance results are on a per-frame basis and corroborate the potential of the algorithm to be the foundation for an intelligent decision support framework that automates the annotation process. The solution can execute annotations in real-time and is significantly more cost-effective than human-only approaches.

## 1. Introduction

Oil and gas operators are governed by regulations that mandate the frequent visual inspections of sub-sea pipelines and platforms in order to assess the condition and risks to these assets. In a typical inspection, a surface vessel deploys a Remote Operating Vehicle (ROV) which is piloted over the pipeline, collecting survey data from multiple sensors/instruments. A typical survey data set comprises of (1) video footage recorded from three camera angles (left/port, center and right/starboard), (2) Inertial Measurement Unit (IMU) data to capture the orientation of the ROV, (3) multi-beam echo sounder data to map the seabed surface and (4) magnetic pipe-tracker to record the pipe location when it is buried below the seabed.

During the inspection, a data coordinator, onboard the surface vessel, provides real-time commentary on survey data and produces initial annotations, identifying events such as pipeline exposure, burial, field joints, anodes, free spans and boulders. The annotation process is prone to human error [1] as data coordinators become fatigued and distracted, leading to missed events or incorrect labeling. After these initial annotations, the video and commentary are subject to Quality Control (QC), either while the survey is ongoing or once completed, creating a bottleneck in the speed of processing and reporting. Furthermore, the speed at which the ROV is piloted is limited by the rate the human can vocalize the presence of an event on audio commentary rather than a limitation of the craft. Automating the survey process enables more consistent, accurate and quicker inspections, while reducing the presence of staff offshore and the concomitant cost and safety risks.

Various vision-based techniques proposed by the Autonomous Underwater Vehicle (AUV) navigation community have primarily focused on pipeline tracking, however, they do not detect and annotate events of interest. Jacobi et al. [2,3], proposed a pipeline tracking method for AUV guidance through the fusion of optical, magnetic and acoustic sensors applied on simulated pipeline data. Narimani et al. [4] proposed a pipeline and cable tracking technique to improve vehicle navigation by converting the images to grey-scale and applying the Hough transformation to determine the angle between vehicle and pipeline; subsequently used as a reference to an adaptive sliding mode controller. Zingaretti et al. [1] developed a real-time vision-based detection system [5] for underwater pipelines using edge-based image processing to detect pipeline contours and a Kalman filter that utilizes the navigation data to reduce the effect of disturbances created by motion. Similarly, Ortiz et al. [6] identified cable contours, in tandem with a linear Kalman filter to predict the contours in the following frame. The same authors presented an alternative approach for tracking using particle filters [7] tested with footage obtained in a water tank. Asif et al. [8] utilized the Bresenham line algorithm to detect noise-free pipeline boundaries and B-Spline to detect active contours subsequently tracked using a Kalman filter.

Sub-sea video footage is particularly challenging to process due to reduced contrast, the presence of suspended particles in the water (e.g., sand, algae), and highly variable illumination. Traditional image processing approaches such as contour determination and their variants, although suitable to localize the edges of the pipeline, require significant feature engineering to detect events such as field joints, free spans and anodes. Sea-life, marine growth, seabed settlements, auxiliary structural elements, breaks on the external pipeline sheathing and alien objects near the pipe are possible sources of confusion in the determination of pipeline contours. Furthermore, it is unclear how these algorithms perform in the absence of the pipeline (when the pipe is buried) or on changes in position and orientation as the ROV maneuvers, both of which result in significant variations of the event appearance in the image plane.

Recently, deep learning approaches have been applied with a similar goal within the power line inspection industry [9,10,11,12]. Nguyen et al. [9] conducted a review on vision-based approaches for power line inspection and the potential role of deep learning. Zhang et al. [10] detected electricity poles in Google Street View Imagery using RetinaNet trained with 1000 annotated images. Jalil et al. [11], utilised Faster-RCNN [13] to detect insulators in drone imagery. Miao et al. [12] implemented a bespoke Single Shot Detector with MobileNet as the backbone to detect insulators. Various applications can also be found for sub-sea imaging. Bonnin-Pascual and Ortiz [14] presented a framework for defect detection on vessels. The approach pre-computed and combined a range of multi-scale normalized feature maps with the use of Gaussian and Gabor pyramid filters. The framework was successfully tested on image mosaics during vessel inspection campaigns. Bonin-Font et al. [15] performed detection, mapping and quantification of Posidonia Oceanica. After initially extracting 168 features from images using a range of kernels and the gray-level co-occurrence matrix, 14 classifiers were trained and compared. Principal Component Analysis (PCA) was applied on the best performing model (Logistic Model Trees) to select the 25 more relevant features and retrain the classifier.

In a continuation of this work, Martin-Abadal et al. [16] created a framework for the semantic segmentation of Posidonia Oceanica. A Deep Fully Convolutional Network was established by VGG16, pre-trained on ImageNet as an encoder, FCN8 as a decoder with Gaussian initialization of its parameters and hyper-parameter tuning. Their model was successfully implemented on a Turbot AUV for the online segmentation of meadows.

In terms of pipeline inspection, Petraglia et al. [17], after initially pre-processing the RGB images, detected pipeline boundaries by firstly filtering edges through Non-Maximum Suppression (NMS) to eliminate horizontal line segments followed by Random Sample Consensus (RANSAC) and Total Least Square (TLS) to group segments. The authors compared two Neural Network (NN) architectures to classify four types of events: inner coating exposure, algae, flange and concrete blankets. The first NN architecture utilizes two convolutional and three fully connected layers, trained on segmented pipelines from the pre-processed images. The second architecture adopted a Multilayer Perceptron (MLP) with a single hidden layer, trained on features extracted from 3-level Wavelet decomposition. The mean and the variance of the wavelet coefficients at each level are then used as features for the neural network, except for the mean of the level-1 low-low coefficients, since the window mean is zero. This feature extraction results in 23 input features from each window. Results led to the conclusion that the convolutional neural network outperforms the MLP, without any need for manual feature extraction.

In this work, transfer learning is harnessed to train a Deep Convolutional Neural Network on raw images of sub-sea pipeline surveys to automatically classify five events (exposure, burial, free span, field joint, anode). The performance evaluation of the proposed framework is conducted on data sets from survey video data obtained from an operational class ROV. The network is configured to perform multi-label image classification which identifies multiple concurrent events in a single frame (for example, exposure and field joint). Data augmentation is used to enhance further the training data sets, facilitating the treatment of the variability embedded within sub-sea images owing to challenges created by dynamic ROV motion, brightness and contrast. Multiple ResNet models of varying depth have been trialed and a ResNet-50 architecture was selected because it balances the trade-off between performance and computation inference time. The ResNet-50 performance yields a high overall Exact Match Ratio and F1-Score of 91.9% and 96.6% respectively on per single frame basis.

## 2. Materials and Methods

**Figure 1:**
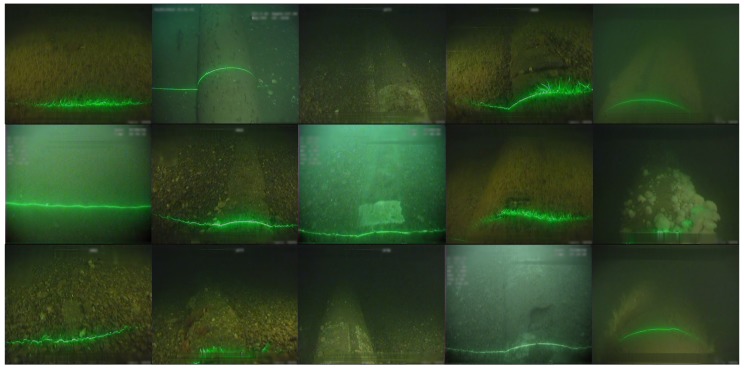
Examples of events in subsea pipeline surveys with varying scene conditions; from left to right: burial, exposure, anode, field joint, free span.

Data sets from two North Sea surveys conducted in 2012 and 2016 covering 201 km and 58 km, respectively were utilized in the development of the automatic annotation system. Each survey recorded three synchronized video feeds (left, center and right) of the pipeline at 25 frames per second. For the purposes of the development, the center camera video only was processed for the following events of interest; examples for various lighting conditions, seabed characteristics and parasites are shown in Figure 1:

- Burial (B): the pipeline is buried underneath the seabed and thus protected.
- Exposure (E): the pipeline is exposed; visible and prone to damage. When the pipeline is exposed to other pipeline features/events become visible:
  – Anode (A): pipeline bracelet anodes are specifically designed to protect sub-sea pipelines from corrosion [18]. Data Coordinators visually recognize anodes by the banding that appears in the orthogonal direction of the pipeline; anodes have no surface vegetation growth.
  – Field joint (FJ): the point where two pipe sections meet and welded together, typically occurring every 12 m. Data coordinators recognize Field Joints due to the depression on the pipeline surface.
  – Free span (FS): pipeline segments that are elevated and not supported by the seabed (either due to seabed erosion/scouring or due to uneven seabed during installation), pose a significant risk to the asset; currents or moving objects (debris, nets and etc.) could damage the pipeline. FS is more apparent on the starboard and port video feeds; the center camera is used to judge the seabed depth against the pipeline.

**Figure 2:**
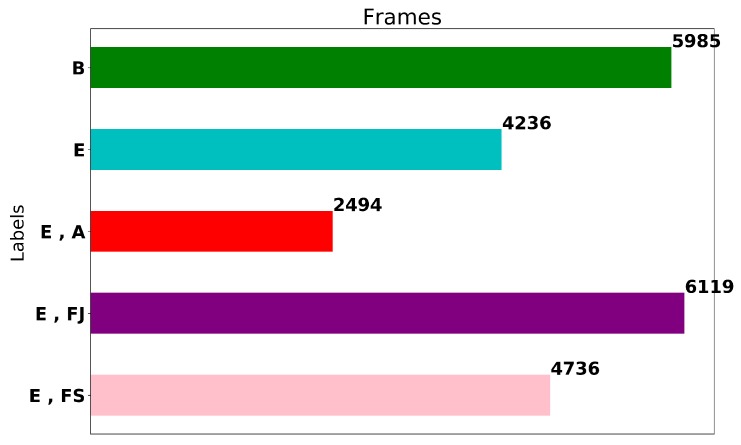
Label distribution of a total 23,570 frames of the complete dataset.

The data set contains event (truthing) annotations created by trained data coordinators. It is important to note that consecutive frames are highly correlated with each other and for that reason still frames were extracted every 10 frames. The frames were labeled using a multi-label annotation approach since events recorded during the pipeline survey are not mutually exclusive. The pipelines are either buried underneath the seabed or exposed and thus visible. However, additional events such as field joints, anodes and free spans are only observable when the pipeline is exposed. This multi-label annotation approach is common practice in the scene classification domain, where images may belong to multiple semantic classes [19]. The label distribution of the extracted frames is shown in Figure 2. The data set contains 23,570 frames in total, consisting of 5985 frames of burial, 4236 frames of exposure, 6119 frames of exposure and field joint, 2494 frames of exposure and anode and 4736 frames of exposure and free span. Note, that all the annotated data (frames and labels) have been checked for annotation correctness three times; one from the data coordinator on the vessel during the execution of the survey, subsequently on-shore by the QC personnel, and finally, after the frames are extracted, by a trained data coordinator who confirmed the annotations through manual inspection.

The first annotation procedure is performed by trained data coordinators on the vessel while the data are captured. For the events, exposure, burial and free span, annotators do not solely rely on video footage, but have information from the Multi-beam Echo which maps the seabed terrain. This makes annotation for these events consistent. The anode and field joint events can be indeed missed during the real-time annotation (although this is unlikely considering the training), this is quality checked onshore (step 2 below). The annotations are verified by a QC Data Coordinators in the office before generating the client report. Routinely, QC data coordinators, have to their disposal annotation data from previous surveys and “as-built” information to corroborate the new survey. This eliminates any missed events, especially the anode and field joint events. Finally, when the frames extracted from the survey data for training and testing datasets, we have performed further manual inspection to ensure any inconsistencies of the labels are corrected.

### 2.1. Model Architecture

A Convolutional Neural Network (CNN) consists of three main types of layers: convolutional, pooling and fully connected. The convolutional layer consists of a set of independent filters which are individually convolved with the input image to generate a series of feature maps as an output [20]. These filters can be adjusted to capture different features of interest within the image. The CNN utilized in the study is based on the ResNet architecture [21], the winner of the ImageNet Large Scale Visual Recognition Challenge 2015 [22]. ResNet is a state-of-the-art architecture that provides enhanced feature extraction capabilities for a wide range of applications, including being a backbone network for implementation of U-Net [23], RetinaNet [24], Faster R-CNN [25] and Mask R-CNN [26]. In this work, the ResNet-50 architecture is used that contains 25.6 M parameters. Other ResNet depths were examined to investigate the trade-off between performance and inference time (Section 6). Typically, a network with a high number of parameters and network depth demands a large training data set to yield acceptable generalization and performance. Creating a training data set of that size is expensive and laborious. An alternative approach is to adopt a transfer learning methodology, where a pre-trained network from a different domain is re-trained on data from the domain of interest (sub-sea pipeline inspection imagery in the present application). The pre-trained ResNet-50 network used is provided by PyTorch [27] trained on the ImageNet data set [22] comprising 1000 image classes.

**Figure 3:**
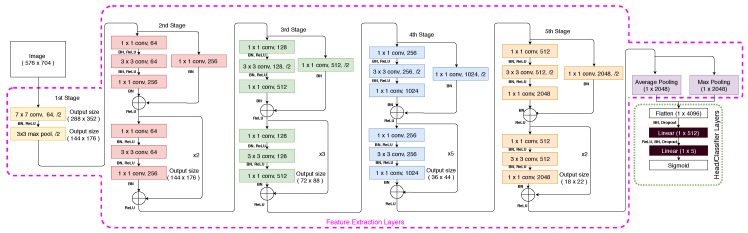
ResNet-50 architecture with modified head.

The ResNet-50 architecture, shown in Figure 3, consists of 5 stages; each stage comprising multiple layers of convolutions, Batch Normalisation [28] and Rectified Linear Unit (ReLU) activations [29] that do not affect the receptive fields of the convolutional layers [29]. More importantly, the ResNet architecture utilizes the concept of skip (or identity) connections between stacked convolutional layers. These shortcut connections mitigate against the vanishing gradient problem on training deep architectures by allowing the gradients to propagate through identity connections. Maintaining the feature extraction layers is a standard methodology for the application of transfer learning. In this case, all the layers in the feature extractor are kept identical with the exception of the final pooling layer. After the fifth stage, the final layer consists of average and max pooling and then features are flattened and concatenated before being fed to two fully connected (linear) layers, with the purpose to reduce the dimensionality of the features and make the dimensions equal to the number of output labels. Furthermore, Batch Normalisation and Dropout layers are introduced between the linear layers to regularise the head/classifier. Note that the last linear layer for the pre-trained network consists of 1000 output neurons to match the number of classes in the ImageNet data set; however, in this application, the output labels are 5 (burial, exposure, free span, field joint, anode) and consequently the last layer is replaced by a linear layer containing five output neurons. The final layer is a Sigmoid activation function to squash network outputs between 0 and 1 independently for each neuron/label [30] using the equation:

(1) $$\hat{y}=\sigma \left(z\right)=\frac{1}{1+e^{-z}},$$

where *z* is the outputs of the last linear layer. The outputs of the network $\hat{y}$ for an image would, therefore, be a vector of five real-valued numbers in the range 0 to 1 (one for each label) which can then be used to compute the sum of binary cross-entropy loss for all labels:

(2) $$L\left(\hat{y},y\right)=-\sum_{i=1}^{C}\left[y_{i}\cdot log\left({\hat{y}}_{i}\right)+\left(1-y_{i}\right)\cdot log\left(1-{\hat{y}}_{i}\right)\right],$$

where *C* is number of labels, *y* is the one-hot encoded target (1 when the label is present in the ground truth data and 0 otherwise) and $y_{i}$ is the element of that vector at location *i*. Similarly, $\hat{y}$ is the predicted vector output of the network and ${\hat{y}}_{i}$ is the element of the vector at location *i* which indicates the confidence level for the corresponding label.

### 2.2. Performance Evaluation Methodology

**Figure 4:**
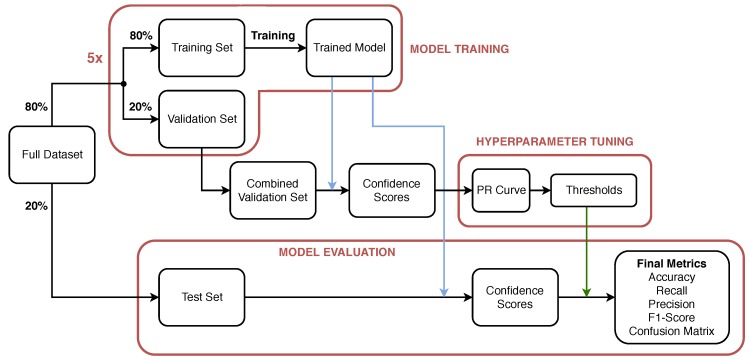
Model training and evaluation process.

The training, validation and testing methodology for the evaluation of the performance of the proposed network is shown in Figure 4. The full data set contains 23,570 frames with annotation according to the label distribution shown in Figure 2. Initially, 20% of the frames in the data set, are selected in a stratified fashion and set aside to be used as a test (keep-out) set and in the evaluation of the performance of the model after training/validation and hyper-parameter tuning. The methodology yields a test set of 4714 frames with label distribution approximately equal to that shown in Figure 2. The remaining 80% (18,856 frames) of the data set is used to perform Monte Carlo Cross-validation [31] with stratified splits of 80/20% i.e., 80% of the data (15,085 frames) is used to train the model and its performance is validated on the remainder 20%; validation set (3771 frames). The process is repeated multiple times (five in this study) to evaluate the variability of the trained models and their performance on the validation sets. After hyper-parameter selection and tuning, the performance of the model is obtained on the test set to ensure representative performance on unseen data.

## 3. Model Training

In practice, training a deep CNN with random initialization for all its weights and biases is challenging, requiring a large data set given the large number of parameters that need to be adjusted. Consequently, a common approach has been adopted, utilizing Transfer Learning [32]. A neural network pre-trained on a large data set of images is used as a starting point. The rationale is that the initial layers of the pre-trained CNN are able to extract features that are generic for image classification tasks; e.g., edge detectors or color blob detectors. In the subsequent layers, network weights need to be fine-tuned to adapt to the specific features of the data set under consideration. In the present study, a deep CNN ResNet-50 [21] pre-trained on the ImageNet data set [33] is implemented (see Figure 3).

The network can be logically divided into two sections; the feature extraction layers (enclosed in purple dashed lines in Figure 3) and the head or classification layers (enclosed in green dashed line in Figure 3). The weights of the feature extraction layers are initialized with the weights obtained from the pre-trained ResNet-50 network distributed with PyTorch 1.2.0 [34], while the head layers are randomly initialized. The Adam optimiser [35,36] is used for training with a mini-batch size of 8 and exponential decay parameters $\beta_{1}$ and $\beta_{2}$ equal to 0.9 and 0.99, respectively. Initially, when the head layers contain random weights, the loss function will yield high errors and thus there is a risk of disturbing the weights of the feature extraction layers when back-propagation is performed. For that reason, a multi-stage training approach is adopted.

In the first stage, training is performed for 4 epochs for only the weights of the last two fully connected layers of the network (shaded in Black in Figure 3), while the weights for all the other layers are frozen; i.e., weights are not updated. Furthermore, cyclic learning rate training [37] is utilized with a maximum learning rate of $10^{-3}$. The cyclic learning rate permits fast convergence and avoids local minima [38] during training. Subsequently, all the layers in the neural network are unfrozen and the network is trained for an additional 2 epochs. For these later epochs, the cyclic learning rate is also adopted, however, different maximum learning rates for the Feature Extraction layers and the head are used; the maximum learning rates are set to $10^{-6}$ and $10^{-4}$, respectively. A lower maximum learning rate is used for the feature extraction layers as their parameters are already well adjusted to extract generic image features. In contrast, the parameters of the head layers need more aggressive adjustment to fit the dataset-specific features. Training is performed on a server equipped with two Nvidia GeForce RTX 2080 Ti, twelve Intel(R) Core(TM) i9-7960X CPU @ 2.80GHz and 128GB RAM.

Given the high capacity of the network, the risk of over-fitting of the training set needs to be evaluated. Two measures are taken to prevent over-fitting: regularisation through weight decay and online data augmentation. For weight decay, the regularisation parameter λ is set to 0.01 for all layers. Online data augmentation is used to increase the variability of the data set and enhance the generalization of the model by limiting over-fitting [39]. A series of transformations are randomly applied to the training data, on every epoch, with probability of 75%, including horizontal flipping, rotation (with maximum angle of 10 degrees), scaling (with maximum variation of 1.05) and lighting alteration (with maximum variation change of 0.1). Data augmentation renders the model more robust and adaptable to the artifacts created, for example, by the motion of the ROV during the survey.

**Figure 5:**
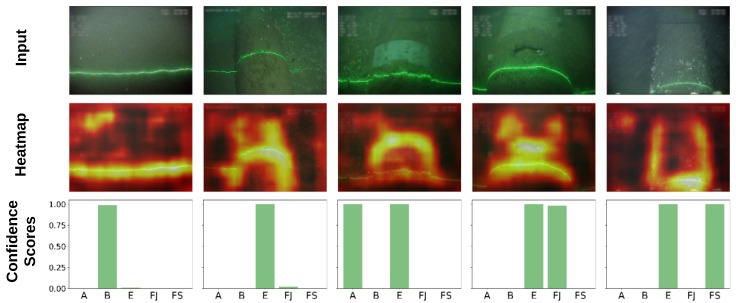
Ground truth label, image, heatmap and predicted confidence scores for the five different event types.

After training, the neural network outputs provide the confidence score for each label. Figure 5 illustrates the confidence scores for each label, for the five selected events; the ground truth labels are shown at the top of each image. In all cases, the trained classifier yields high confidence scores (bottom bar chart) for these classes. The straight laser line observed when the pipeline is buried is the most relevant feature of the burial class, judging by the corresponding heat map (image on the middle row). When the pipeline is exposed, the model tends to focus on both its cylindrical shape and the curved nature of the laser line. In cases where other pipeline elements are visible, the model uses additional features to correctly classify the image. For example, for field joints, the unstructured depression/hole in the middle of the pipeline becomes a relevant feature; for anodes, the dominant feature is the characteristic white bracelet; for free spans, the most important feature is the well-defined edges of the pipeline resulting from its elevation with respect to the seabed.

The examples presented in Figure 5 have been intentionally extracted from the two different surveys and at different positions within each survey to highlight the large variety of image scenes. On consideration of the entire data set, these variations include differences in color (green, brown, grey), type of seabed (sand or gravels), vegetation (low or high) and distance and orientation of the ROV with respect to the seabed. The more variety the training set contains, the better the generalization of the trained classifier will be.

## 4. Hyperparameter Tuning and Model Validation

After training, when an image is presented to the network input, the network output, after the final Sigmoid activation layer, is a vector with the degrees of confidence on whether or not each label is associated with the input image. In order to perform the final prediction and decide whether or not each label is associated with the input image, a threshold must be defined to make the output discrete; 1 if confidence score exceeds the threshold, 0 otherwise. The threshold can be either defined using a common value for all labels or defining five thresholds, one for each class/label [40,41]. Here, five separate thresholds are defined, one for each label to permit optimal performance per class. The selection of the thresholds is a means to adjust the sensitivity of the model for each label. Low thresholds will lead to high detection sensitivity at the expense of false positives (FP), while high thresholds will reduce FPs at the expense of missed Positives [42]. The five threshold values constitute the model hyper-parameters and Precision–Recall Curves are used to determine optimal values, as illustrated in Figure 4. Precision–Recall curves are used in binary, and thus multi-label, classification to define a cut-off point (threshold) on the output confidence that the classifier assigns to each label and is commonly used in unbalanced data sets [43]. Note that the definition of the optimal thresholds is executed using solely the validation set, only containing images unseen during the training phase.

The evaluation of performance in multi-label learning is more challenging than in traditional single class settings, because each event can be associated with multiple labels simultaneously. In particular the following metrics are of interest:

(3) $$Accuracy=\frac{TP+TN}{TP+FP+TN+FN}$$

(4) $$Recall=\frac{TP}{TP+FN}$$

(5) $$Precision=\frac{TP}{TP+FP}$$

(6) $$F1-Score=\frac{2\cdot Precision\cdot Recall}{Precision+Recall}.$$

In this application, when metrics for a specific label are reported, the problem is reduced to a binary classification One-vs-Rest [44]. For instances where aggregate performance is reported, then the “micro” average [45] is computed. The exception is for aggregate accuracy, in which case, successful classifications counts are used only after all the labels have been identified correctly, commonly also known as “Exact Match Ratio” (EMR), a stricter metric, compared to average accuracy. Formally, the EMR is defined as:

(7) $$ExactMatchRatio=\frac{1}{n}\sum_{i=1}^{n}𝟙(y_{i}=\hat{y_{i}}),$$

where $𝟙(y_{i}=\hat{y_{i}})$ is the indicator function equal to 1 only when every element in the vector $y_{i}$ is equal to every element in $\hat{y_{i}}$ and *n* is the number of input samples. Note that for a binary classification (i.e., individual labels), this reduces to accuracy.

**Figure 6:**
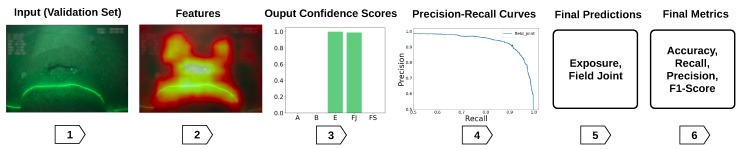
Steps for evaluating model’s performance: (**1**) validation set, (**2**) feature extraction, (**3**) classifier, (**4**) precision–recall curves for optimal thresholds selection, (**5**) applying optimal thresholds, (**6**) comparison with ground truth.

Steps 1–4 in Figure 6 illustrate the process followed to obtain optimal threshold selection on the validation set. Note that due to five-fold Monte Carlo cross-validation, five different models are trained, one for each validation fold. The predictions obtained from the five independent models on the five different validation folds are concatenated and used to determine the optimum set of thresholds/hyper-parameters. Precision–recall curves can then be generated to evaluate the performance of the classifier at increasing values of confidence score thresholds. For each threshold value, the final set of predictions is evaluated against the corresponding ground truths at the individual label basis to identify each prediction as true positive (TP), false positive (FP), true negative (TN) or false negative (FN). The precision and recall of the classifier are then calculated using Equations (4) and (5) (step 4 in Figure 6). The optimum threshold is defined as the point that achieves the best balance between precision and recall, and therefore corresponds to the closest point to the top right corner on the graph (coordinate (1,1)). The strategy to define the optimal threshold was selected because, in this application, it is equally important to maximize precision and recall to provide the maximum F1-score.

**Figure 7:**
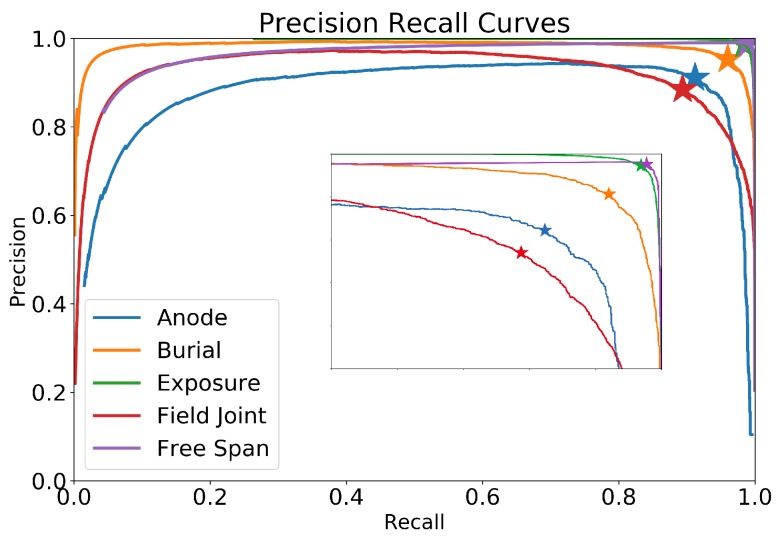
Precision–recall curves for all labels. The inset shows a zoomed version of the top right corner.

Applying the methodology for the five event types (anode, burial, exposure, field joint and free span), results in the precision–recall curves shown in Figure 7. The optimal thresholds are at the locations depicted by the star (“*”) carets in the graph and yield thresholds for each event type, summarised in Table 1.

Using the optimal thresholds identified from hyper-parameter tuning, the performance metrics (Equations (3)–(7)) for each model in their corresponding validation fold is shown in Table 2.

Similarly, the average performance of the five models for each event type is shown in Table 3 along with the standard deviation for each metric. Field joints are the most challenging class with the lowest F1-score of 88.9%, expected given that such events are often difficult to distinguish due to the subtle features. On the other extreme, free spans and exposures show high performance, with F1-score of 98.8% and 98.5%, respectively. The aggregate F1-score (micro-average) is 96%.

## 5. Model Performance on Test Set

**Figure 8:**
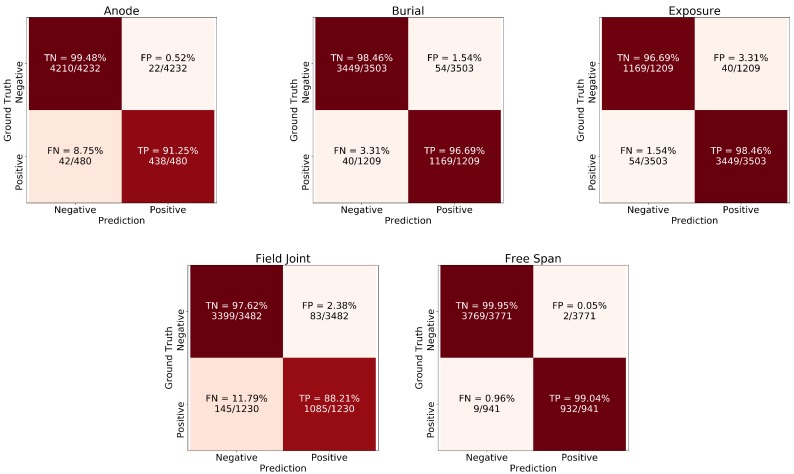
Confusion matrices on the test set for each class; anode, burial, exposure, field joint and free span.

In order to ensure that thresholds are not biased to the validation set, the final model performance evaluation is carried out on a previously unseen (keep-out) test set (Figure 4); i.e., the images that have not been used for either training nor validation or hyper-parameter tuning. The cross-validation has yielded five different models and the model selected for final testing is the one that provides the highest F1-Score viz. the model of the third fold, shown in bold in Table 2. Figure 8 shows the confusion matrices for each label, obtained using the final model on the test set. Each label is considered positive if it is present in the image frame and negative otherwise. The confusion matrices show the absolute number of frames and the percentage of TN, FP, FN, TP. For instance, the total number of frames in the test set is 4712 frames with 480 frames associated with the label “anode” and 4232 are not. From the 480 frames that are labeled as “anode” (positive frames), 438 (91.25%) have been correctly identified by the model (TP) and 42 (8.75%) have been missed (FN). In terms of FP, 22 frames have been incorrectly identified as anodes out of 4232 i.e., a false positive rate of 0.52%.

From the confusion matrices, the Field Joints are the most challenging label with a miss rate of 11.79% and false positive rate of 2.38%. FJ misclassifications can be attributed to visual artifacts in the imagery, for example, small rocks or vegetation. It is worth noting that this is the classifier performance on a single frame basis; when the classifier is applied on a video stream with 25 fps, the probability that these artifacts appearing in all frames is reduced and as a consequence, the probabilities of a missing event or incorrect identification reduces, respectively. The performance of the network on per label basis is summarised in Table 4. Overall, the accuracy (exact match ratio) of the network is 91.9% with F1-Score of 96.6%.

## 6. Effect of Model Size

Identical evaluation performance was carried out for ResNet models with 18, 34, 101 and 152 layers (in addition to 50). The resultant performance metrics, on the test set, for each model size are summarised in Table 5. As the model complexity and capacity increase, the F1-Scores initially improves until the ResNet-50 architecture. Further increases in the number of layers (i.e., 101 and 152), result in performance degradation. Larger models have a tendency to overfit faster. This is likely to occur given the training parameters are kept identical; i.e., the number of epochs, regularisation coefficients, learning rates and etc. and altering these parameters may be necessary to achieve optimal prediction accuracy. Even though larger networks have the potential to achieve better F1-Score, as the number of layers increases, the number of parameters increase significantly along with the inference times. Note that inference time reports in Table 5 are the average computation time over 100 frame predictions; i.e., 100 forward passes. For the deeper networks, the inference time is marginally within the bounds of real-time operation. From these results, the ResNet-50 model is selected as it provides the best performance with inference time within the bounds of real-time operation.

## 7. Conclusions

A ResNet-50 deep convolutional neural network is employed to automatically detect and annotate five sub-sea survey events; anode, exposure, burial, field joint, and free span relying exclusively on the center video feed of an ROV. To minimize the challenging demands on the scope of the training data, a transfer learning approach is adopted where the feature extraction layers of the network are initialized using the weights of a network pre-trained on ImageNet. The head of the network is adjusted to permit multi-label classification yielding the identification of events appearing concurrently in the video frames. Subsequently, the developed network is re-trained on 23,570 images extracted from real survey data. Several network depths were tested and the ResNet-50 network was selected to balance the trade-off between performance and inference time. The network has been evaluated on a test keep-out set to measure its ability to generalize. The framework achieves an exact match ratio (i.e., all labels identified correctly) of 91.9% and a F1-Score ‘micro’-average of 96.6%. The most challenging class to detect are Field Joints which have been detected with accuracy of 95.1% and F1-Score of 90.4%, respectively. The metrics are obtained on a single-frame basis and the proposed network is able to classify frames within 23.6 ms on an NVIDIA GeForce RTX 2080 Ti GPU, effectively executing real-time classification of video streams at 25 fps. Results, along with the real-time operation of the network demonstrate that automatic video annotation has the potential to increase the speed of survey execution, increase the consistency of annotation and reduce the demand on off-shore personnel, benefiting health and increasing safety. Future work will investigate the benefits in combining predictions from consecutive frames and the fusion of the video data with multi-beam echo, pipe-tracker instrumentation to improve annotation performance.

## Figures and Tables

**Table 1:** Optimum label-based thresholds for the validation set.

Event	Anode	Burial	Exposure	Field Joint	Free Span
**Threshold**	0.357	0.367	0.632	0.542	0.430

**Table 2:** Aggregate performance of the five models, one for each fold.

Fold #	Exact Match Ratio	Precision	Recall	F1-Score
1	0.907	0.958	0.961	0.960
2	0.890	0.949	0.956	0.953
3	0.920	0.972	0.961	0.967
4	0.914	0.962	0.967	0.964
5	0.899	0.954	0.958	0.956

**Table 3:** Metrics with optimal thresholds on the validation set.

	Threshold	Accuracy		Recall		Precision		F1-Score	
Event		Average	Std	Average	Std	Average	Std	Average	Std
**Anode**	0.357	0.981	0.006	0.910	0.028	0.912	0.046	0.911	0.028
**Burial**	0.367	0.978	0.001	0.959	0.011	0.953	0.013	0.956	0.004
**Exposure**	0.632	0.978	0.001	0.984	0.004	0.986	0.003	0.985	0.001
**Field Joint**	0.542	0.942	0.008	0.893	0.020	0.885	0.024	0.889	0.015
**Free Span**	0.430	0.995	0.002	0.988	0.002	0.988	0.013	0.988	0.007
**Aggregate**		0.906	0.011	0.961	0.004	0.959	0.008	0.960	0.005

**Table 4:** Test set performance of individual labels and aggregate.

Event	Threshold	Accuracy	Precision	Recall	F1-Score
Anode	0.357	0.986	0.952	0.912	0.931
Burial	0.367	0.980	0.955	0.966	0.961
Exposure	0.632	0.980	0.988	0.984	0.986
Field Joint	0.542	0.951	0.928	0.882	0.904
Free Span	0.430	0.997	0.997	0.990	0.994
**Aggregate**		**0.919**	**0.972**	**0.960**	**0.966**

**Table 5:** Test set performance of different ResNet model sizes.

Network	# Parameters	Inference Time (ms)	Exact Match Ratio	Precision	Recall	F1-Score
**ResNet-18**	11,706,949	17.7	0.872	0.945	0.947	0.946
**ResNet-34**	21,815,109	20.8	0.903	0.953	0.966	0.960
**ResNet-50**	25,617,477	23.6	0.919	0.972	0.960	0.966
**ResNet-101**	44,609,605	31.2	0.916	0.956	0.973	0.965
**ResNet-152**	60,253,253	39.1	0.833	0.931	0.927	0.929
